# Pregnant women with periodontal disease: can complete blood count be useful?

**DOI:** 10.1590/1414-431X2024e14097

**Published:** 2025-03-03

**Authors:** P.C.S. Sá, A.P.N. Godoi, G.C.S. Bernardes, N.A. Almeida, L.S. Nogueira, M.G. Carvalho, M. Barros-Pinheiro

**Affiliations:** 1 Universidade Federal de São João del-Rei, Campus Centro-Oeste Dona Lindu, Divinópolis, MG, Brasil; 2 Departamento de Análises Clínicas e Toxicológicas, Faculdade de Ciências Farmacêuticas, Universidade Federal de Alfenas, Alfenas, MG, Brasil; 3 Departamento de Análises Clínicas e Toxicológicas, Faculdade de Farmácia, Universidade Federal de Minas Gerais, Belo Horizonte, MG, Brasil

**Keywords:** Pregnancy, Gingivitis, Periodontitis, Blood cell count, Pregnancy hematologic complications, Hematologic tests

## Abstract

Prenatal care is of fundamental importance and must be carried out by a multidisciplinary healthcare team, including dental care, as several changes and complications affecting the oral cavity may occur during pregnancy. This was a cross-sectional study that aimed to analyze the hematological profile of pregnant women with and without periodontal disease (PD). Data were obtained by consulting medical and dental records, which were stratified into two subgroups: pregnant women with PD (n=107) and pregnant women without PD (n=42). Study variables were related to PD, sociodemographic, clinical, and laboratory characteristics. Data were collected from the complete blood count and the following indices were calculated: neutrophil-to-lymphocyte ratio (NLR), platelet-to-lymphocyte ratio (PLR), lymphocyte-to-monocyte ratio (LMR), derived NLR (dNLR), systemic inflammation response index (SIRI), aggregate index of systemic inflammation (AISI), and systemic immune-inflammation index (SII). The mean age in both subgroups was 27 years. Pregnant women with less education had more PD. Mean corpuscular volume was significantly higher in pregnant women with PD, probably a reflection of folate deficiency. White blood cell and lymphocyte counts were significantly higher in pregnant women with periodontitis, possibly reflecting an inflammatory process caused by bacterial invasion of the periodontium with systemic repercussions. This study reinforces the need for a multidisciplinary team, including a dentist, in prenatal care, to lower the risk of complications for the mother and child.

## Introduction

Pregnancy is a transition phase in a woman's life cycle, which is part of the natural process of human development. Physiological, physical, psychological, and even pathological transformations can occur (1). In view of this, prenatal care is fundamental and must be carried out by a multidisciplinary health team. In addition to medical follow-up, imaging, and laboratory tests, it is important to have specific dental care since several complications may occur during pregnancy that affect the oral cavity (2).

The multidisciplinary follow-up of pregnant women should aim to impart knowledge for a safe delivery, which is important for maternal and fetal health (2,3). In oral health, prenatal guidance on preventive and educational dental care, such as plaque control, healthy eating, and oral hygiene, should be clearly provided throughout the pregnant woman's dental treatment (2,3). Poor oral hygiene habits can favor premature birth and low birth weight (3).

Estrogen and progesterone levels are increased during pregnancy and affect oral physiology, modifying the balance of the oral cavity and influencing the oral health of pregnant women (2,4). Therefore, hormonal changes can intensify preexisting problems in the oral cavity, because pregnancy itself would not cause such complications (5). Even if such alterations are not enough to cause oral pathology, they become relevant in this period and deserve a careful look by the dentist. As reviewed by da Silva et al. (5), systemic changes during this period can influence the periodontium but are not sufficient to cause the pathology itself.

Important modifications occur in the maternal system during pregnancy (6-8). These include a progressive increase in plasma volume, fibrinolytic activity, absolute number and concentration of leukocytes in the peripheral blood, and coagulation factors. An increase in the absolute number of red blood cells is also observed although with a decrease in their concentration, in addition to a progressive reduction in the platelet count, which usually remains within normal limits (1). Based on the premise that both periodontal disease (PD) and pregnancy provoke an inflammatory state with systemic repercussions, the monitoring of pregnant women through complete blood counts plays an important role in clinical care.

Complete blood count is a test that is inexpensive, easy to perform, reproducible, available, and very informative (1). In view of this, the present study investigated the hematological profile of pregnant women with and without PD in the second and third trimesters of pregnancy, since several physiological changes in the hematological system that occur in the first trimester would be confounding factors in the study. In the first trimester there is an increase in blood volume ranging from 40 to 50% with a consequent increase in plasma volume and blood cells. Red cell mass also increases in the first trimester and, to a lesser extent, in subsequent trimesters. This initial increase is around 30%, which is less pronounced compared to the plasma volume. This unbalanced ratio leads to hemodilution, a necessary condition for the ideal transport of oxygen to the fetus (9,10).

The relationship between PD and its impact on the peripheral blood of pregnant women is still not clear. Therefore, it is interesting to investigate the impact of PD on pregnant women since it is an inflammatory disease with potential repercussions on peripheral blood. On the other hand, according to Mor (11) and Mor et al. (12), pregnancy is a pro-inflammatory and anti-inflammatory condition, depending on the stage of gestation. The first and third trimesters have a pro-inflammatory environment, whereas the second trimester has an anti-inflammatory environment. Thus, the sum of the pro-inflammatory factors of PD and pregnancy provides an important clinical perspective worth investigating. In this context, this study aimed to investigate the impact of PD on the hematological profile of pregnant women.

## Material and Methods

This was a cross-sectional study carried out in the city of Divinopolis, MG, Brazil, involving 310 pregnant women in the second and third trimesters of pregnancy who were attended at the Basic Health Units. To avoid possible errors and biases in the collection of data from pregnant women, pre-testing, training, and assessment of examiners, validation of the instrument used for data collection, and a pilot study were carried out before the study. Data from medical records and laboratory tests of 494 pregnant women invited to participate in the study were assessed. Of this total, 310 pregnant women agreed to participate and the eligible participants signed the Informed Consent Form. Participants responded to the face-to-face interview using a standardized instrument and underwent a clinical oral examination performed by duly trained dentists. Participants with oral alterations were then referred to the public health network for dental treatment.

Of the 310 pregnant women who agreed to participate in the study, 61 were excluded because they were in the first trimester of pregnancy and 100 because their respective blood cell counts were not available in the Municipal Medical Records System. The remaining 149 pregnant women were stratified into two subgroups as follows: a) pregnant women with periodontal disease (n=107), including only gingivitis (n=78) and periodontitis (n=29); and b) pregnant women without periodontal disease (n=42) as controls, matched for age with those with PD.

**Figure 1:**
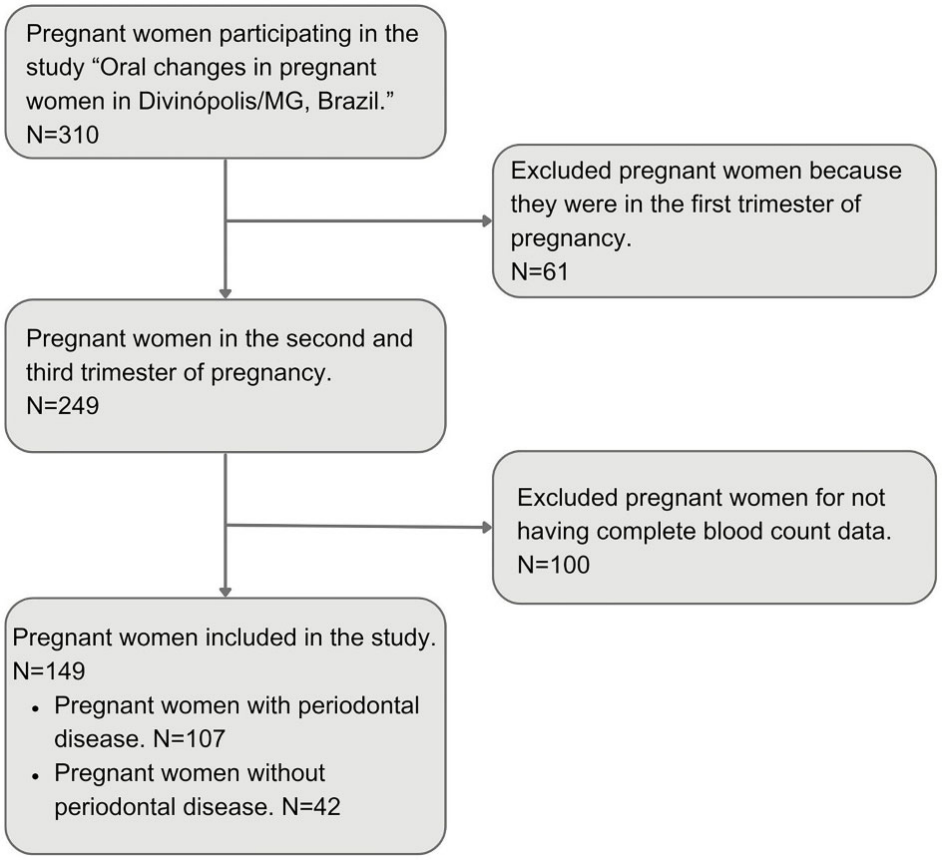
Flowchart of the study conducted from September 2020 to July 2021, in Divinopolis, MG, Brazil.

The diagnosis and classification of PD followed the recommendations of the American Academy of Periodontology and the European Federation of Periodontology (2018). Inclusion criteria were women in the second and third trimesters of pregnancy who were aged 18 years or older and whose medical and dental records were available in the health system. Pregnant women who were cognitively unable to answer the questionnaire and had no medical and dental clinical data and blood results were excluded. The study workflow can be seen in Figure 1.

Pregnant women with PD were classified according to the degree of gingivitis or periodontitis. Descriptive data analysis was performed using frequency distribution and means±SD for parametric data and median and interquartile range for nonparametric data. Data normality was tested using the Kolmogorov-Smirnov method. For comparison between groups, Student's *t-*test was performed for parametric data and the Mann Whitney test for nonparametric data. Categorical variables were compared using chi-squared test. The analyses were performed using the Statistical Package for the Social Sciences^®^ software (version 19.0, IBM, USA), with a significance level of P<0.05.

The study was conducted in accordance with the Declaration of Helsinki and approved by the Ethics and Research Committee of the UFSJ/CCO (CAAE: 20648719.3.0000.5545).

## Results

Participants in both groups had a mean age of 27 years. A rather curious finding that the number of pregnant women with PD who required high-risk prenatal care was significantly higher than those who did not have PD (P=0.02), as shown in Table 1. Other characteristics such as marital status, level of education, and family income were not significantly different between the two groups. However, a strong tendency for PD was found in women with lower family income compared to those with higher family income (P=0.09).

**Table 1:** Characteristics of pregnant women in the second and third trimesters of pregnancy with and without periodontal disease attended at the Basic Health Units of Divinopolis, MG, Brazil, from September 2019 to July 2021.

Variable	Pregnant women without periodontal diseasen=42 (%)	Pregnant women with periodontal diseasen=107 (%)	P-value
Age (years, mean±SD)	27 (±6.5)	27 (±6)	0.97^b^
Current BMI (median and 25 and 75 percentiles)	27 (25-33)	27 (24-32)	0.88^c^
BMI at first prenatal visit (median and Q1 and Q3)	25 (21-28)	25 (22-30)	0.3^c^
Marital status, n (%)			
Single	15 (35.7)	39 (36.4)	
Married	27 (64.3)	68 (63.6)	0.94^a^
Education (n=148), n (%)			
Until 9 years old	5 (11.9)	23 (21.7)	
Until 10 to 12 years old	25 (59.5)	74 (69.8)	
Over 12 years old	12 (28.6)	9 (8.5)	0.13^a^
Family income (n=138), n (%)			
Until 1 minimum wage	14 (33.3)	47 (49.0)	
2 or more minimum wages	28 (66.6)	49 (51.0)	0.09^a^
High risk prenatal care, n (%)			
No	35 (83.3)	68 (63.6)	
Yes	7 (16.7)	39 (36.4)	0.02^a^
Other pregnancies, n (%)			
No	21 (50.0)	41 (38.3)	
Yes	21 (50.0)	66 (61.7)	0.20^a^

^a^Chi-squared; ^b^Student's *t*-test; ^c^Mann-Whitney test; BMI: body mass index.

Blood cell count data were compared according to the gingival clinical condition observed in the same period of time. No difference was found in hematological variables between pregnant women in the second trimester with and without PD. On the contrary, pregnant women in the third trimester with PD had significantly higher mean corpuscular volume (MCV) values than those without PD (92.99±5.50 *vs* 89.83±6.43, respectively, P=0.016). A trend towards higher red cell distribution width (RDW) values in women with PD in the third trimester of pregnancy was also observed compared to those without PD in the same gestational stage [12.80 (11.90-14.00) versus 12.00 (11.57-13.50), respectively, P=0.059] (Table 2).

**Table 2:** Hematological variables of pregnant women in the second and third trimesters of pregnancy with and without periodontal disease attended at the Basic Health Units of Divinopolis, MG, Brazil, from September 2019 to July 2021.

Variable	Pregnant women without periodontal disease(second trimester)	Pregnant women with periodontal disease(second trimester)	P-value		Pregnant women without periodontal disease(third trimester)	Pregnant women with periodontal disease(third trimester)	P-value
Gestational age (weeks)	24 (18-25)n=11	20 (16-25)n=44	0.459^b^		35 (32-37)n=31	33 (30-37)n=63	0.191^b^
Red blood cells (x10^6^/mm^3^)	4.45 (±0.49)n=11	4.27 (±0.59)n=44	0.379^a^		4.36 (±0.49)n=30	4.15 (±0.53)n=63	0.074^a^
Hemoglobin (g/dL)	13.24 (±1.29)n=11	12.89 (±1.16)n=44	0.388^a^		12.73 (±1.36)n=30	12.63 (±1.51)n=63	0.746^a^
Hematocrit (%)	40.51 (±4.59)n=11	39.11 (±3.81)n=44	0.301^a^		39.02 (±3.96)n=30	38.40 (±4.80)n=63	0.540^a^
MHC (pg)	29.76 (±2.39)n=10	30.42 (±2.51)n=44	0.431^a^		29.65 (28.17-31.45)n=30	30.70 (29.40-31.90)n=63	0.068^b^
MCV (fL)	91.16 (±5.75)n=11	92.99 (±5.40)n=44	0.325^a^		89.83 (±6.43)n=30	92.99 (±5.50)n=63	0.016^a^
MCHC (%)	32.73 (±1.15)n=10	32.72 (±1.21)n=44	0.986^a^		32.90 (32.07-33.40)n=30	32.90 (32.00-33.60)n=63	0.761^b^
RDW (%)	11.90 (11.70-13.00)n=11	12.45 (11.62-13.40)n=44	0.643^b^		12.00 (11.57-13.50)n=30	12.80 (11.90-14.00)n=63	0.059^b^
White blood cells (×10^3^/mm^3^)	9.34 (±2.82)n=11	9.41 (±2.54)n=44	0.977^a^		8.72 (±2.79)n=30	8.31 (±2.04)n=63	0.427^a^
Neutrophils(×10^3^/mm^3^)	5.25 (4.10-7.51)n=11	5.43 (4.50-8.17)n=44	0.768^b^		5.47 (±2.42)n=30	5.31 (±1.81)n=63	0.722^a^
Lymphocytes(×10^3^/mm^3^)	2.40 (2.20-2.71)n=11	2.24 (2.00-2.67)n=44	0.288^b^		2.25 (1.97-3.02)n=30	2.11 (1.83-2.40)n=63	0.083^b^
Monocytes(×10^3^/mm^3^)	0.59 (±0.21)n=11	0.60 (±0.19)n=44	0.447^a^		0.57 (0.45-0.64)n=30	0.50 (0.39-0.62)n=63	0.187^b^
Eosinophils(×10^3^/mm^3^)	0.10 (0.07-0.23)n=11	0.13 (0.08-0.20)n=44	0.908^b^		0.16 (0.10-0.29)n=29	0.14 (0.08-0.26)n=63	0.389^b^
Basophil(×10^3^/mm^3^)	0.06 (±0.02)n=11	0.08 (±0.05)n=43	0.390^a^		0.06 (0.04-0.09)n=29	0.05 (0.03-0.07)n=62	0.146^b^
Platelets(×10^3^/mm3)	231 (214-247)n=11	215 (196-268)n=44	0.474^b^		240 (±66)n=29	231 (±45)n=63	0.442^a^

^a^Student's *t*-test; ^b^Mann-Whitney test. Data are reported as means±SD or median and interquartile range. MHC: Mean corpuscular hemoglobin; MCV: mean corpuscular volume; MCHC: mean corpuscular hemoglobin concentration; RDW: red cell distribution width.

Table 3 shows the comparison of pregnant women in the second and third trimesters of pregnancy with and without PD in relation to indices derived from complete blood count parameters. None of the comparisons showed a significant difference.

**Table 3:** Indexes derived from parameters of the complete blood count of pregnant women in the second and third trimesters of pregnancy with and without periodontal disease attended at the Basic Health Units of Divinopolis, MG, Brazil, from September 2019 to July 2021.

Variable	Pregnant women without periodontal disease(second trimester)	Pregnant women with periodontal disease(second trimester)	P-value	Pregnant women without periodontal disease(third trimester)	Pregnant women with periodontal disease(third trimester)	P-value
NLR	2.50 (±1.28)n=11	2.64 (±1.08)n=44	0.701^a^	2.37 (±1.16)n=30	2.67 (±1.23)n=63	0.273^a^
PLR	96.33 (±27.43)n=11	99.71 (±31.78)n=44	0.748^a^	101.83 (85.33-122.69)n=29	110.14 (86.07-129.51)n=63	0.217^b^
LMR	4.96 (3.21-5.86)n=11	4.01 (3.10-5.25)n=44	0.383^b^	4.09 (3.33-5.56)n=30	4.34 (3.07-5.46)n=63	0.984^b^
dNRL	1.84 (±0.84)n=11	1.92 (±0.69)n=44	0.769^a^	1.74 (±0.76)n=30	1.91 (±0.79)n=63	0.330^a^
SIRI	0.90 (0.60-2.34)n=11	1.46 (0.80-2.25)n=44	0.514^b^	1.35 (0.72-1.91)n=30	1.26 (0.83-2.05)n=63	0.873^b^
AISI	218 (129-541)n=11	348 (205-529)n=44	0.436^b^	257 (205-435)n=29	269 (176-414)n=63	0.886^b^
SII	610 (±390)n=11	603 (±287)n=44	0.945^a^	493 (379-649)n=29	561 (384-720)n=63	0.253^b^

^a^Student's *t*-test; ^b^Mann-Whitney test. Data are reported as means±SD or median and interquartile range. NLR: neutrophil-to-lymphocyte ratio; PLR: platelet-lymphocyte ratio; LMR: lymphocyte-to-monocyte ratio; dNLR: derived neutrophil-to-lymphocyte ratio; SIRI: systemic inflammation response index; AISI: aggregate index of systemic inflammation; SII: systemic immune-inflammation index.

Global white blood cell and lymphocyte count values were significantly higher in pregnant women in the second trimester according to the degree of gingival inflammation [10.61±2.34 for periodontitis and 8.91±2.49 for gingivitis, P=0.042; 2.67 (2.19-3.49) for periodontitis and 2.16 (1.88-2.47) for gingivitis, P=0.007, respectively]. In addition, pregnant women in the third trimester with only gingivitis had higher mean corpuscular hemoglobin concentration (MCHC) values compared to pregnant women with periodontitis [33.10 (32.50-33.70); 32.65 (31.37-33.00), respectively, P=0.039] (Table 4).

**Table 4:** Hematological variables of pregnant women in the second and third trimesters of pregnancy with gingivitis or periodontitis attended at the Basic Health Units of Divinopolis, MG, Brazil, from September 2019 to July 2021.

Variable	Pregnant women with gingivitis(second trimester)	Pregnant women with periodontitis(second trimester)	P-value	Pregnant women with gingivitis(third trimester)	Pregnant women with periodontitis(third trimester)	P-value
Gestational age (weeks)	20.00(16.00-25.00)n=31	22.00(17.50-26.00)n=13	0.332^b^	34.00(30.00-37.00)n=47	30.00(28.25-36.00)n=16	0.177^b^
Red blood cells(×10^3^/mm^3^)	4.20 (±0.62)n=31	4.45 (±0.50)n=13	0.207^a^	4.16 (±0.50)n=47	4.13 (±0.63)n=16	0.858^a^
Hemoglobin (g/dL)	12.76 (±1.25)n=31	13.20 (±0.87)n=13	0.253^a^	12.71 (±1.44)n=47	12.38 (±1.73)n=16	0.442^a^
Hematocrit (%)	38.67 (±4.09)n=31	40.16 (±2.91)n=13	0.241^a^	38.49 (±4.61)n=47	38.14 (±5.48)n=16	0.800^a^
MHC (pg)	30.67 (±2.45)n=31	29.94 (±2.67)n=13	0.385^a^	31.10(29.40-32.30)n=47	30.30(28.72-31.60)n=16	0.265^b^
MCV (fL)	93.38 (±5.30)n=31	92.08 (±5.72)n=13	0.471^a^	92.98 (±5.41)n=47	92.99 (±5.92)n=16	0.996^a^
MCHC (%)	32.84 (±1.23)n=31	32.43 (±1.15)n=13	0.305^a^	33.10(32.50-33.70)n=47	32.65(31.37-33.00)n=16	0.039^b^
RDW (%)	12.50(11.80-13.40)n=31	12.10(11.00-13.40)n=13	0.425^b^	12.80(11.60-14.00)n=47	13.10(12.22-14.05)n=16	0.487^b^
White blood cells (×10^3^/mm^3^)	8.91 (±2.49)n=31	10.61 (±2.34)n=13	0.042^a^	8.20 (±1.99)n=47	8.65 (±2.20)n=16	0.453^a^
Neutrophils(×10^3^/mm^3^)	5.43(4.07-8.18)n=31	7.11(5.09-8.28)n=13	0.310^b^	5.31 (±1.91)n=47	5.32 (±1.54)n=16	0.984^a^
Lymphocytes(×10^3^/mm^3^)	2.16(1.88-2.47)n=31	2.67(2.19-3.49)n=13	0.007^b^	2.06(1.76-2.63)n=47	2.26(1,87-2.51)n=16	0.320^b^
Monocytes(×10^3^/mm^3^)	0.59 (±0.17)n=31	0.62 (±0.24)n=13	0.665^a^	0.45(0.39-0.63)n=47	0.54(0.47-0.62)n=16	0.155^b^
Eosinophils(×10^3^/mm^3^)	0.12(0.08-0.20)n=31	0.13(0.11-0.22)n=13	0.263^b^	0.14(0.08-0.26)n=47	0.13(0.09-0.28)n=16	0.764^b^
Basophils(×10^3^/mm^3^)	0.07 (±0.03)n=30	0.10 (±0.07)n=13	0.060^a^	0.05(0.03-0.07)n=47	0.060.03-0.08)n=16	0.879^b^
Platelets(×10^3^/mm^3^)	211(196-268)n=31	232(184-284)n=13	0.700^b^	227 (±47)n=47	242 (±36)n=16	0.234^a^

^a^Student's *t*-test; ^b^Mann-Whitney test. Data are reported as means±SD or median and interquartile range. MHC: mean corpuscular hemoglobin; MCV: mean corpuscular volume; MCHC: mean corpuscular hemoglobin concentration; RDW: red cell distribution width.

All the indices derived from complete blood count parameters were also evaluated according to the degree of gingival inflammation. The lymphocyte to monocyte ratio (LMR) showed a significant difference between the groups of pregnant women with gingivitis and periodontitis only in the second trimester, with higher values in those with periodontitis [3.78 (2.81-4.84); 5.00 (3.85-6.98), P=0.033, respectively] (Table 5). On the contrary, although not significant, the platelet to lymphocyte ratio (PLR) tended to be lower in pregnant women with periodontitis compared to those with gingivitis only at the same gestational stage [85.37 (±30.52); 105.72 (±30.80), respectively, P=0.052].

**Table 5:** Indexes derived from parameters of complete blood count of pregnant women in the second and third trimesters of pregnancy with gingivitis or periodontitis attended at the Basic Health Units of Divinopolis, MG, Brazil, from September 2019 to July 2021.

Variable	Pregnant women with gingivitis(second trimester)	Pregnant women with periodontitis(second trimester)	P-value	Pregnant women with gingivitis(third trimester)	Pregnant women with periodontitis(third trimester)	P-value
NLR	2.72 (±1.10)n=31	2.47 (±1.05)n=13	0.481^a^	2.71 (±1.27)n=47	2.54 (±1.14)n=16	0.627^a^
PLR	105.72 (±30.80)n=31	85.37 (±30.52)n=13	0.052^a^	110.14 (84.78-130.74)n=47	109.50 (97.68-120.94)n=16	0.975^b^
LMR	3.78 (2.81-4.84)n=31	5.00 (3.85-6.98)n=13	0.033^b^	4.30 (2.95-5.79)n=47	4.39 (3.33-4.66)n=16	0.746^b^
dNLR	1.94 (±0.69)n=31	1.85 (±0.70)n=13	0.692^a^	1.95 (±0.81)n=47	1.79 (±0.76)n=16	0.488^a^
SIRI	1.71 (±0.99)n=31	1.54 (±1.07)n=13	0.611^a^	1.51 (±1.19)n=47	1.37 (±0.61)n=16	0.648^a^
AISI	348 (200-575)n=31	348 (180-391)n=13	0.690^b^	266 (161-266)n=47	310 (235-398)n=16	0.394^b^
SII	608 (±269)n=31	591 (±339)n=13	0.857^a^	572 (377-721)n=47	530 (391-822)n=16	0.975^b^

^a^Student's *t*-test; ^b^Mann-Whitney test. Data are reported as means±SD or median and interquartile range. NLR: neutrophil-to-lymphocyte ratio; PLR: platelet-to-lymphocyte ratio; LMR: lymphocyte-to-monocyte ratio; dNLR: derived neutrophil-to-lymphocyte ratio; SIRI: systemic inflammation response index; AISI: aggregate index of systemic inflammation; SII: systemic immune-inflammation index.

## Discussion

It is widely known that pregnancy causes important changes in the hematological profile. Thus, it has been reported that mainly in the first trimester, the white blood cell count increases as the pregnancy progresses, with segmented and immature neutrophils (also called bands) being the most frequent cells (13,14). According to the same authors, while the number of neutrophils increases, the number of lymphocytes drops significantly, causing immunological suppression, which is necessary for the woman's body to hold the fetus. Only after the tenth week of pregnancy, due to the elevation of estrogen and progesterone hormones, changes occur in the metabolism of organs and systems, also leading to alterations in the periodontium of pregnant women (10). Therefore, the present study was carried out with women already in the second and third trimesters of pregnancy.

As reported by Cauchi et al. (15), a significant proportion of pregnant women have abnormal red blood cell volume distribution, particularly in the second half of pregnancy, resulting from an increased proportion of microcytic cells in 8% of patients and macrocytosis in 67% of patients. These findings are in line with our findings of significantly higher MCV and a tendency towards increased RDW in pregnant women in the third gestational trimester with PD compared to those without PD. This may be justified, at least partially, by a mild folate deficiency. Likewise, iron deficiency in some pregnant women could also be contributing to the tendency towards an increase in RDW, that is, anisocytosis, which is frequently observed in pregnant women.

Folic acid acts as a coenzyme in the metabolism of amino acids, synthesis of purines and pyrimidines, and formation of DNA and RNA, and is therefore essential for cell division (16). Its lack can lead to disturbances in erythropoiesis (3). It can be speculated that the consumption of folic acid by bacteria associated with periodontal diseases in pregnant women may contribute to the increase in MCV. Although studies on the correlation between bacteria associated with such periodontal diseases and folate consumption were not found, this hypothesis cannot be excluded, as oral cavity infections can be caused by a complex microbial community with about 700 bacterial species (17).

During pregnancy, the consumption of folates increases by five to ten times. Inadequate folate intake leads to folate deficiency, which is initially manifested in the blood count by the presence of some macrocytes in the blood smear, an increased MCV or with a tendency to increase (3). Although MCV values were within the normal range in our study, they were significantly greater in pregnant women with PD in the third trimester. This finding is already suggestive of a slight folic acid deficiency among these women. Women in this group also had a greater frequency of high-risk prenatal care during the follow-up. These factors are certainly linked to a possible nutritional deficiency, especially of folic acid. Despite the clear link between higher levels of IL-6, LDH, and C-reactive protein and the presence of chronic periodontitis in pregnant women (18), which could theoretically affect erythropoiesis, López et al. (19) did not observe significant changes for either MCV or HCM in pregnant women with PD. Based on the data observed in our study, the risk of megaloblastic anemia in some pregnant women with PD is very small considering that MCV values did not reach 100 fL. This anemia is characterized by the presence of giant erythroblasts in the bone marrow that will originate macrocytic erythrocytes with an increase in MCV (above 98 fL) and MCH (above 38 g/dL), but with MCHC within the normal range (30 to 35 g/dL) (10,20).

In this study, the other components of the erythrogram did not show significant differences between pregnant women with and without PD neither in the second nor in the third trimester. Other studies have reported an irrelevant correlation between hemoglobin level and chronic periodontitis in individuals of both the sexes (21). On the other hand, Antony and Khan (6) concluded that chronic periodontitis is associated with a drop in hemoglobin concentration proportional to the severity of the oral disease. Also, other studies have observed a tendency towards a reduction in hematocrit values in patients with periodontitis compared to healthy people, but this decrease was not significant (19,22).

A significant difference was observed for MCHC and for global white blood cell and lymphocyte counts. The MCHC index was significantly higher in pregnant women with gingivitis in the third trimester compared to pregnant women with periodontitis. This fact may partly reflect the impact of this inflammatory disease on the bone marrow, causing a slight reduction in the synthesis of hemoglobin, which is probably not happening in pregnant women with only gingivitis. According to Antony et al. (6), chronic periodontitis is associated with hemoglobin concentration and, therefore, hemoglobin levels tend to fall proportionally to the severity of periodontitis. The MCHC depends on hemoglobin levels and hematocrit value (MCHC = Hb/Hto. × 100) and is influenced by the MCV (that is, the globular volume of erythrocytes). In the present study, a significant increase in MCV (tendency to macrocytosis) was observed in pregnant women with PD in the third trimester, which certainly results in some degree of increase in the hematocrit value. Thus, in line with the MCHC formula, the significantly lower MCHC found in pregnant women with periodontitis can be partially explained by the hematocrit value. As previously mentioned (6), chronic periodontitis can lead to a reduction in hemoglobin levels, which might also contribute to the significantly reduced MCHC in pregnant women with periodontitis compared to those with gingivitis. On the other hand, global white blood cell and the absolute lymphocyte counts were significantly higher in pregnant women with periodontitis in the second semester. This result may be paradoxical, as the second trimester is considered, according to Mor (11), an anti-inflammatory phase, while the third trimester is pro-inflammatory. Theoretically, an inflammatory process could be exacerbated in the last trimester. These data may contribute to the literature, as they indicate that pregnant women in the second trimester need to be given more attention if they have periodontitis, since the inflammatory process may increase even more, requiring extra care for the pregnant woman. In this context, it is important to emphasize that the inflammatory process increases insulin resistance, which may harm maternal and fetal health (11).

Based on our data, it can be inferred that periodontitis is associated with an inflammatory process and systemic repercussions, which was shown by a significantly higher number of white blood cells compared to the group with only gingivitis. Previous studies have reported a relationship between white blood cell count and periodontal disease in individuals with periodontitis (23,24). Patients with moderate and severe periodontitis had a significantly higher number of lymphocytes and total white blood cells compared to healthy individuals (23). Sharma et al. (24) observed a correlation between PD severity and global white blood cell count in patients with chronic periodontitis with a decrease in the number of these cells after treatment. In this context, it is important to emphasize that periodontitis is a chronic disease that can have a systemic effect on pregnant women, maximizing the existing inflammatory potential. On the other hand, gingivitis tends to be limited to a local effect that may not trigger a systemic inflammatory reaction (25).

None of the blood count indices showed a significant difference between pregnant women with and without PD in the studied gestational trimesters. However, when these indices were compared between pregnant women with gingivitis or periodontitis, the LMR was significantly higher in women with periodontitis in the second trimester of pregnancy. The literature is scarce about the causes of hematological changes in pregnant women and in individuals with PD. Indices extracted from peripheral blood cell counts have not yet been evaluated in the context of dental alterations. It is possible that an acute inflammatory process in the periodontium results in an important alteration in the peripheral blood. However, more studies are needed to reveal the actual role of these indices in more severe cases in dental practice.

As previously mentioned, Pejcić et al. (23) reported that patients with moderate and severe periodontitis had significantly higher numbers of lymphocytes and total white blood cells compared with healthy subjects. Thus, it can be suggested that some pregnant women in this study presented PD in more advanced stages resulting in lymphocytosis and implying an increase in LMR. In clinical practice, systemic inflammation can be directly indicated by changes in peripheral blood attested by immune and inflammatory cell counts (routine cells such as neutrophils, lymphocytes, and monocytes, in addition to platelets) and by acute phase proteins such as C-reactive protein, whose dosage was not determined in the present study. These new indices, which are widely used for assessing the prognosis of COVID-19 patients, combine the above cell types and reflect the complex interaction between immune and inflammatory cells (26-28).

It is known that periodontitis progresses with increasing age (29), but the average age of pregnant women involved in the present study was the same in both groups (27 years), and within the ideal age range for gestation, which is between 18 and 28 years old (22). No significant difference was identified in body mass index (BMI), marital status, family income, and number of pregnancies between the groups of pregnant women with and without PD. However, it is known that some of these variables can interfere in the development of some diseases, such as periodontitis (30). Oral health is often neglected, especially during pregnancy. Good general health, including oral health, requires adequate working conditions, in addition to access to education, housing, and leisure (31). As a result, people who have a disadvantaged financial condition will also have less access to basic health services in general. Financial limitations are the main obstacles to accessing health services. However, such difficulties can also be influenced by cultural issues, such as fear of going to the dentist (31).

In our study, pregnant women with PD had lower education compared to those without PD. It is possible that this factor outweighs all the other factors mentioned above due to its influence on the behavior of individuals in society. Oral hygiene habits are influenced not only by economic problems, but also by cultural and educational issues. Raising awareness of the importance of flossing and the main precautions to be taken for proper oral hygiene, in addition to carrying out preventive and early treatments and not only treatment in acute situations, would undoubtedly help to minimize problems related to periodontal diseases (31,32).

Another finding of this study was that pregnant women with PD were followed up in high-risk prenatal care more frequently compared to pregnant women without PD. This difference was statistically significant and may be related to economic, cultural, and educational aspects (4).

The innovative nature of this study was the use of the complete blood count as an additional tool in the follow-up of pregnant women regarding oral care. The inclusion of complete blood counts and their proper interpretation in dental practice can also have other benefits outside the context of PD, as it may detect changes suggestive of diseases that impact dental procedures. As dental alterations can have unfavorable systemic repercussions, special care must be taken to ensure that general health conditions of the pregnant woman and consequently also of her fetus are optimally maintained. Particularly, the oral health of pregnant women is of paramount importance in preventing premature birth, premature rupture of membranes and low birth weight, as well as for the well-being of these women at such a special moment in their lives.

On the other hand, the wide range of reference values for the complete blood count parameters makes interpretation difficult, as in most cases no data prior to pregnancy and periodontal disease are not available for comparison. This is a limitation of the complete blood count that exists alongside many advantages and benefits for prenatal care.

Despite the advantages of this study in terms of knowledge about the possible implications of PD on the hematological status of pregnant women, the lack of data regarding folic acid supplementation is a limitation of this investigation.

In short, the present study evaluated the socioeconomic and hematological profiles of pregnant women with and without PD. As expected, PD was more prevalent in pregnant women with lower education and socioeconomic. Regarding hematological changes in the red blood cells, erythrocyte MCV was significantly higher in pregnant women with PD in the third trimester, probably a reflection of folate deficiency. As for white blood cells, the total number of leukocytes and lymphocytes was significantly higher in pregnant women with periodontitis, possibly reflecting a chronic inflammatory process caused by bacterial invasion of the periodontium with systemic repercussions. The study hypothesis was confirmed, that is, “periodontal alterations in pregnant women can potentiate changes in the typical hematological parameters of pregnancy”.

Our study reinforces the need for a multidisciplinary healthcare team, including a dentist in prenatal care, given that pregnancy is known to be a period with many physical, psychological, and even pathological changes. This would ensure adequate healthcare for mother and child.
